# Gait Parameters Measured from Wearable Sensors Reliably Detect Freezing of Gait in a Stepping in Place Task

**DOI:** 10.3390/s21082661

**Published:** 2021-04-10

**Authors:** Cameron Diep, Johanna O’Day, Yasmine Kehnemouyi, Gary Burnett, Helen Bronte-Stewart

**Affiliations:** 1 Department of Neurology and Neurological Sciences, Stanford University School of Medicine, Stanford, CA 94305, USA; cdiep@stanford.edu (C.D.); odayj@stanford.edu (J.O.); ykehn97@stanford.edu (Y.K.); garyb@stanford.edu (G.B.); 2 Department of Bioengineering, Stanford University, Stanford, CA 94305, USA; 3 Department of Biomedical Data Science, Stanford University, Stanford, CA 94305, USA; 4 Department of Neurosurgery, Stanford University School of Medicine, Stanford, CA 94305, USA

**Keywords:** Parkinson’s disease, wearables, inertial measurement unit, sensors, freezing of gait

## Abstract

Freezing of gait (FOG), a debilitating symptom of Parkinson’s disease (PD), can be safely studied using the stepping in place (SIP) task. However, clinical, visual identification of FOG during SIP is subjective and time consuming, and automatic FOG detection during SIP currently requires measuring the center of pressure on dual force plates. This study examines whether FOG elicited during SIP in 10 individuals with PD could be reliably detected using kinematic data measured from wearable inertial measurement unit sensors (IMUs). A general, logistic regression model (area under the curve = 0.81) determined that three gait parameters together were overall the most robust predictors of FOG during SIP: arrhythmicity, swing time coefficient of variation, and swing angular range. Participant-specific models revealed varying sets of gait parameters that best predicted FOG for each participant, highlighting variable FOG behaviors, and demonstrated equal or better performance for 6 out of the 10 participants, suggesting the opportunity for model personalization. The results of this study demonstrated that gait parameters measured from wearable IMUs reliably detected FOG during SIP, and the general and participant-specific gait parameters allude to variable FOG behaviors that could inform more personalized approaches for treatment of FOG and gait impairment in PD.

## 1. Introduction

Freezing of gait (FOG), often defined as a feeling of one’s feet being “glued” to the floor [1,2], is a debilitating phenomenon in Parkinson’s disease (PD) that negatively impacts quality of life and can lead to falls, serious injury, or even death [3,4,5,6]. FOG is a challenging phenomenon to objectively measure in the clinic and laboratory [7], but several tasks, such as 360-degree turning in place [8], the turning and barrier course (TBC) [9], and stepping in place (SIP) [10], have been developed to safely and reliably elicit FOG.

The SIP task is a safe and powerful tool for studying FOG. However, clinical, visual identification of FOG during SIP is subjective and time consuming, and automatic, computerized FOG detection during SIP currently requires measuring vertical ground reaction forces on dual force plates [10]. These force plates are large, immobile, and only used in laboratories and clinics. Instead, wearable inertial measurement unit sensors (IMUs) are small and portable, allowing for use in daily life. IMUs have been used to characterize and predict FOG during several tasks [9,11,12,13,14,15,16,17,18,19,20,21,22,23,24,25,26,27,28,29,30,31,32,33,34,35,36,37], and many of these IMU-based FOG detection schemes rely on statistical and machine learning methods [9,11,12,13,14,15,16,17,18,19,20,21,22]. For example, IMUs measured kinematic data during straight walking and the TBC [9,37]. A statistical model developed using this data revealed that certain gait parameters—stride time, swing angular range, asymmetry, and arrhythmicity—reliably predicted FOG during the TBC [9,37]. No study has investigated if IMUs can detect FOG during SIP, and no statistical model has been developed to determine which gait parameters most reliably predict FOG during SIP.

In this study, we examined whether FOG elicited during SIP could be reliably detected using kinematic data measured from two IMUs worn on the shanks. We aimed to develop a statistical model using multiple gait features to determine which gait parameters best predicted FOG during SIP and gain insight into potential personalized approaches for treatment of FOG and gait impairment in PD.

## 2. Materials and Methods

### 2.1. Participants

Data were obtained from 10 participants (5 female) with clinically established PD at the Stanford Movement Disorders Center. All participants gave their written informed consent to participate in this study, which was approved by the Food and Drug Administration and the Stanford University School of Medicine Institutional Review Board. Participants were tested off therapy (medication and/or deep brain stimulation). Long-acting dopaminergic medication was withdrawn over 24 h prior to testing (48–72 h for extended-release dopamine agonists), and short-acting dopaminergic medication was withdrawn over 12 h prior to testing. Deep brain stimulation was turned off at least 2 min before testing. A certified rater performed the Unified Parkinson’s Disease Rating Scale Part III (UPDRS III) [38] and the Freezing of Gait Questionnaire (FOG-Q) [39].

### 2.2. Experimental Protocol

The SIP task is a validated task that elicits FOG and consists of repetitive alternating stepping in place at a self-selected pace on dual force plates [10]. Participants begin by standing at rest. At a “go” cue, participants step in place for 100 seconds, ending with a “stop” cue. All participants are harnessed during the task as a safety measure. Data from one trial of SIP per participant were analyzed in this work.

### 2.3. Data Acquisition

External videos of the SIP task were recorded on an encrypted clinical iPad (Apple Inc., Cupertino, CA, USA) for offline video review. Ground reaction forces were captured at 1000 Hz with two force plates (Bertec, Columbus, OH, USA). Participants were instrumented with IMUs (APDM Opals, APDM, Inc., Portland, OR, USA) on the lateral side of the shanks so that one axis of each sensor was aligned with the sagittal plane of the participant. Triaxial gyroscope and accelerometer signals from the IMUs were sampled at 128 Hz. The data were filtered using a zero-phase 8th order low pass Butterworth filter with a 9 Hz cut-off frequency, and principal component analysis was used to align the angular velocity with the sagittal plane.

**Figure 1:**
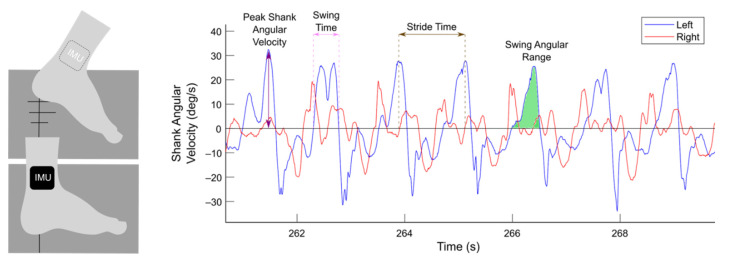
Gait parameters extracted from wearable inertial measurement unit sensors (IMUs). Participants performed the stepping in place task on dual force plates (dark gray). Two IMUs were mounted on the lateral side of the shanks, and shank angular velocities in the sagittal plane from the left (blue) and right (red) legs were measured. Gait parameters, such as peak shank angular velocity (purple), swing time (pink), stride time (brown), and swing angular range (green), were extracted from shank angular velocity data.

Data gathered from the IMUs were used to measure and calculate a total of eight gait parameters: peak shank angular velocity, stride time, swing angular range, swing time, swing time coefficient of variation (CV), asymmetry, arrhythmicity, and freeze index. Using the angular velocity measured by the IMUs (Figure 1), individual steps were identified as positive peaks in the left and right sagittal shank angular velocity plot. These peaks represent the absolute maxima shank angular velocity in a step cycle and were marked as steps only if they exceeded a minimum threshold of 10 deg/s [9]. Stride time was defined as the time between two successive positive peaks on the angular velocity plot. Swing angular range was calculated as the area under a peak on the sagittal angular velocity plot. Swing time was computed as the time between swing phase initiation and end, as determined by zero-crossings on the angular velocity plot. Swing time CV was defined as the standard deviation of swing time divided by average swing time over a window of the previous six steps. Asymmetry was defined as 100 × |ln(SSWT/LSWT)|, where SSWT and LSWT correspond to the leg with the shortest and longest mean swing time over a window of the previous six steps. Arrhythmicity was calculated as the average stride time CV of the previous three stride times of the left and right leg. Freeze index was defined as the power in the freezing band (3–8 Hz) divided by the power in the gait band (0.5–3 Hz) [40]. Analysis of these gait parameters was performed in MATLAB (version 9.8, The MathWorks Inc., Natick, MA, USA). All gait parameters were scaled and normalized to have zero mean and unit variance.

### 2.4. FOG Detection

All participants exhibited at least one freezing episode during the SIP task, as identified by offline video review.

Ground-truth labels of freezing episodes were automatically identified from force plate data by a previously validated, computerized algorithm [10]. Briefly, the algorithm used external video recordings as ground truth labels of freezing episodes and detected freezing episodes from vertical ground reaction forces measured by dual force plates. A freezing episode was defined as a period when the participant’s feet did not fully lift off from the force plates (i.e., when the vertical forces did not reach 100% or 0% of bodyweight) or as an abnormally long interval between two steps [10]. These ground-truth labels of freezing and non-freezing episodes were used to create and balance the training and testing sets used to build the models, as discussed in the following section.

Data from the force plates and IMUs were synchronized by inducing a force or rapid acceleration that was detected by both systems, allowing for the validation of kinematic, sensor-based FOG detection with kinetic, force plate-based FOG detection. IMUs were synchronized with each other by Motion Studio software (APDM, Inc., Portland, OR, USA).

### 2.5. Logistic Regression Models of FOG during SIP

Binomial logistic regression models were developed to calculate the probability that a given step during SIP was considered part of a freezing episode. A threshold of 0.5 was set such that a given step was categorized as part of a freezing episode when the probability that it was part of a freezing episode exceeded 50%. Ground-truth, binary labels of freeze steps (non-freeze = 0, freeze = 1) from the previously validated algorithm [10] and eight gait parameters (peak shank angular velocity, stride time, swing angular range, swing time, swing time CV, asymmetry, arrhythmicity, and freeze index) were used to build the binomial logistic regression models in R (version 4.0.2, R Core Team (2020)).

To develop the general model, the entire data set of eight gait parameters from all 10 participants was scaled to have zero mean and unit variance, balanced so that there was an equal number of freeze steps as non-freeze steps, and shuffled. 75% of each participant’s data were pooled into the training data set, while the remaining 25% of each participant’s data were pooled into the testing data set. The general model was then trained and tested on the respective data sets using 10-fold cross validation.

To develop the participant-specific models, the data set of eight gait parameters was first separated by participant. Each participant’s data set was then scaled to have zero mean and unit variance, balanced so that there was an equal number of freeze steps as non-freeze steps, and shuffled. The participant-specific models were trained on 75% of the respective participant’s data and tested on the remaining 25% of the participant’s data using 10-fold cross validation.

Model performance was evaluated by calculating the area under the receiver operating curve (AUC), accuracy (number of correct predictions divided by the total number of predictions), sensitivity (number of freezes identified by the model divided by the total number of true freezes), and specificity (number of non-freezes identified by the model divided by the total number of true non-freezes).

## 3. Results

### 3.1. Participants

Among the 10 participants (5 female), average age was 62.5 ± 8.8 years, average disease duration was 10.3 ± 3.5 years, average off therapy UPDRS III score was 38.4 ± 11.2, and average FOG-Q question 3 (FOG-Q3) score was 2.1 ± 1.5 (Table 1).

### 3.2. Gait Parameters Measured by Wearable IMUs Detected FOG during SIP

**Figure 2:**
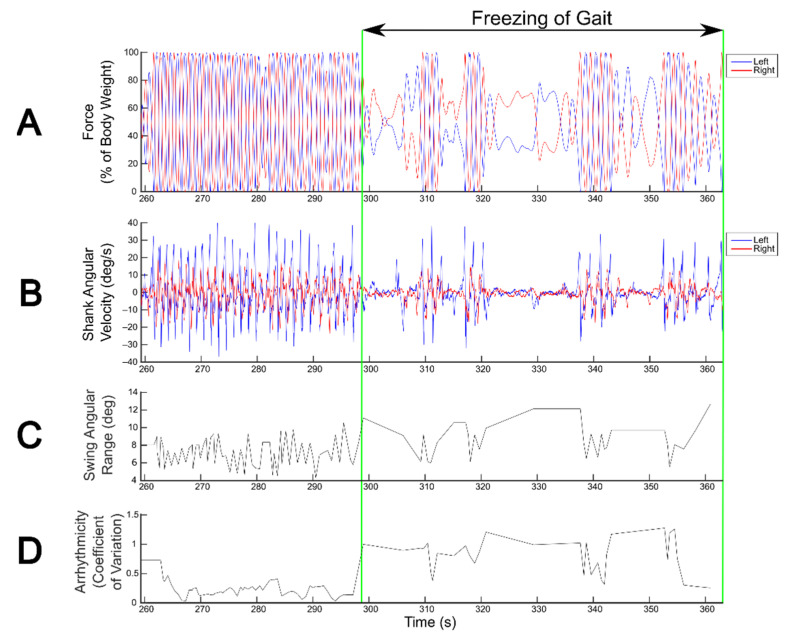
Detection of freezing of gait (FOG) during the stepping in place (SIP) task. FOG detection during SIP from kinetics derived from vertical force measured by dual force plates (**A**) and from kinematics derived from shank angular velocity measured by wearable inertial measurement unit sensors (**B**). Blue and red traces correspond to the left and right legs, respectively. Swing angular range (**C**) and arrhythmicity (**D**) calculated from shank angular velocity show visually detectable differences between freezing and non-freezing episodes.

Gait parameters measured from wearable IMUs reliably detected FOG during SIP, capturing changes in gait metrics, such as swing angular range and arrhythmicity, that distinguish freezing from non-freezing episodes (Figure 2).

### 3.3. A General Model Revealed That Three Gait Parameters Best Predicted FOG during SIP Overall

Logistic regression models based on kinematic data measured from wearable IMUs detected FOG during SIP on a step-by-step basis. The general model, tested on all participants’ data, achieved an AUC value of 0.81, accuracy of 0.84, sensitivity of 0.86, and specificity of 0.81 (Table 2).

**Figure 3:**
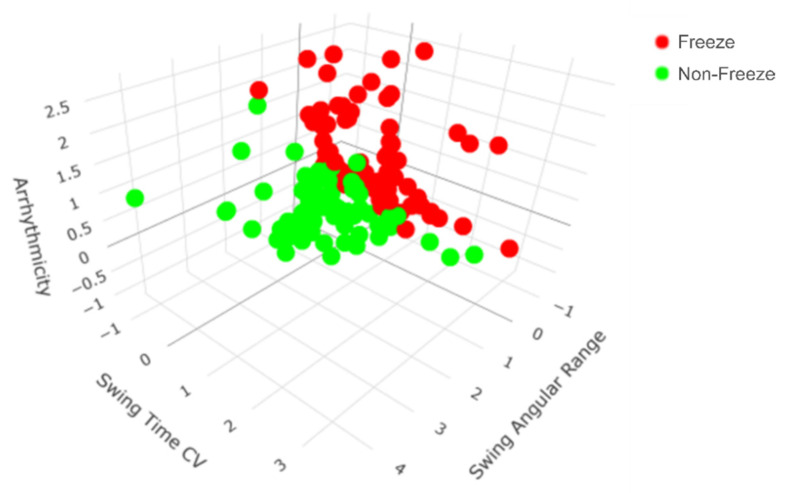
All steps from all participants mapped according to the top three general predictors of freezing of gait (FOG) during the stepping in place (SIP) task. The general model revealed that arrhythmicity, swing time coefficient of variation (CV), and swing angular range together were the most robust predictors of FOG during SIP. There is a clear separation between freeze (red) and non-freeze (green) steps during SIP.

The general model determined that the three most robust predictors of FOG during SIP were arrhythmicity (coefficient = 1.076), swing time CV (coefficient = 0.894), and swing angular range (coefficient = −0.06), with an intercept of 0.017 (Figure 3). Peak shank angular velocity (coefficient = −0.006) also distinguished freeze from non-freeze steps during SIP with a smaller coefficient. The probability that a step during SIP was considered part of a freezing episode was calculated using the aforementioned, normalized gait parameters, denoted as *X_AR_*, *X_SWCV_*, *X_SA_*, and *X_AV_*, respectively:

(1) $$P\left(FOG\right)=\frac{1}{1+e^{-\left(0.017+1.076*X_{AR}+0.894*\;X_{SWCV}-0.06*X_{SA}-0.006*X_{AV}\right)}}$$

### 3.4. Participant-Specific Models Revealed Varying Sets of Gait Parameters That Best Predicted FOG during SIP and Could Outperform the General Model for Some Participants

**Figure 4:**
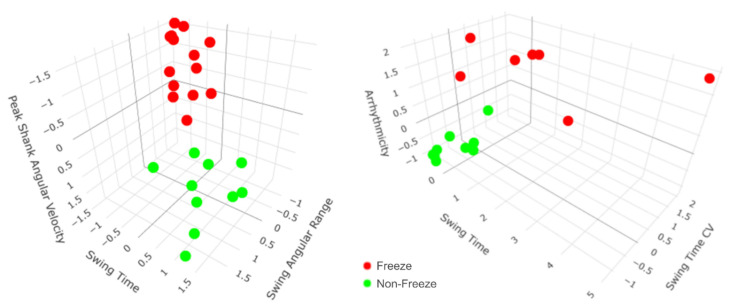
All steps from participants 1 and 2 mapped according to their top three participant-specific predictors of freezing of gait (FOG) during the stepping in place (SIP) task. Participant-specific models revealed that varying sets of gait parameters best predicted FOG during SIP for each participant. For example, participant 1′s most robust predictors of FOG were a combination of peak shank angular velocity, swing time, and swing angular range (**left**), while participant 2′s most robust predictors of FOG were a combination of arrhythmicity, swing time, and swing time coefficient of variation (CV) (**right**). For both participants, there is a clear separation between freeze (red) and non-freeze (green) steps during SIP.

Although arrhythmicity, swing time CV, and swing angular range together were the most robust predictors of FOG in general (Figure 3), the participant-specific models revealed that varying sets of gait parameters best predicted FOG for each participant (Table 3). For example, the most robust predictors of FOG for participant 1 were a combination of peak shank angular velocity, swing time, swing angular range, and arrhythmicity, while the most robust predictors of FOG for participant 2 were a combination of arrhythmicity, swing time, swing time CV, and asymmetry (Figure 4). External video recordings of participants’ SIP trials showed that each participant exhibited one, some, or all of the following FOG behaviors: complete akinesia (i.e., no observable motion of the legs), small stepping (i.e., festination or shuffling), trembling in place (i.e., small movements of the leg or knee with no effective stepping motion), and slow stepping (i.e., sticky feet).

Although the general model achieved an overall AUC of 0.81 (Table 2), it performed with variable accuracy among the participants, ranging from 0.60 to 1.00. Participant-specific models, on the other hand, achieved equal or higher accuracies than the general model for 6 out of the 10 participants. Sensitivity was equal or higher for 8 out of the 10 participants, and specificity was equal or higher for 7 out of the 10 participants (Table 4). Values of 1.00 for accuracy, sensitivity, and specificity were likely the result of small training and testing sets used to build the models.

## 4. Discussion

This study determined that gait parameters measured from wearable IMUs reliably detected FOG during the SIP task. A general, logistic regression model (AUC = 0.81) determined that three gait parameters together were overall the most robust predictors of FOG during SIP: arrhythmicity, swing time CV, and swing angular range. Participant-specific models revealed varying sets of gait parameters that best predicted FOG for each participant and demonstrated equal or better performance for 6 out of the 10 participants.

### 4.1. Gait Parameters Measured from Wearable Inertial Sensors Detected FOG in PD

Automatic FOG detection during SIP on dual force plates has been previously validated [10], but these force plates are large, immobile, and only used in laboratories and clinics. Instead, wearable IMUs are small and portable, allowing for use in daily life. This study determined that IMUs worn on the shanks reliably detected FOG elicited during SIP by capturing changes in gait parameters, such as swing angular range and arrhythmicity, that distinguish freezing from non-freezing episodes. These results extend the accessibility of the SIP task for FOG assessment to the home environment.

### 4.2. A General Model for FOG Detection during SIP Determined That Three Gait Parameters Were Overall the Most Robust Predictors of FOG

Logistic regression models based on kinematic data measured from wearable IMUs detected FOG during SIP on a step-by-step basis. The general model achieved an AUC value of 0.81, accuracy of 0.84, sensitivity of 0.86, and specificity of 0.81, which are similar to other IMU-based FOG detection algorithms [8,9,11,12,13,14,15,16,17,18,19,20,21,22,23,24,25,26,27,28,29,30,31,32,33,34,41].

The general model revealed that three gait parameters together were overall the most robust predictors of FOG during SIP: arrhythmicity, swing time CV, and swing angular range. The probability that a step was considered part of a freezing episode increased as arrhythmicity and swing time CV increased and as swing angular range decreased. This is consistent with the abnormal gait pattern generation thought to cause FOG. The positive correlation between arrhythmicity and FOG supports previous findings that people who experience FOG exhibit greater arrhythmicity and bilateral dyscoordination of left-right stepping than people who do not experience FOG [10,42,43,44]. This relationship can also be explained by the impaired temporal control of gait cycles during FOG [42,45,46], further reinforcing the rhythmicity and bilateral coordination required while performing the SIP task [10]. In addition, increased step time variability and cadence often precede and accompany FOG [42,45,46]. Our model also captured increased swing time CV during FOG in SIP, consistent with the finding that gait variability increases in PD [47] and prior to FOG [48,49]. Finally, gait impairment preceding freezing episodes has been characterized by reduced joint angle ranges in the hip, knee, and ankle [45]. Similarly, our model revealed that FOG during SIP was often characterized by decreased swing angular range.

### 4.3. Participant-Specific Models Highlight the Opportunity for Personalized Approaches for the Treatment of FOG and Gait Impairment in PD

In general, arrhythmicity, swing time CV, and swing angular range were the three most robust predictors of FOG during SIP. However, the participant-specific models revealed that varying sets of gait parameters best predicted FOG for each participant. For example, the most robust predictors of FOG for participant 1 were a combination of peak shank angular velocity, swing time, swing angular range, and arrhythmicity, while the most robust predictors of FOG for participant 2 were a combination of arrhythmicity, swing time, swing time CV, and asymmetry. The differences between sets of predictive gait parameters suggest that each participant’s FOG may be caused by differing levels of gait impairment, possibly due to variable disease durations and severity [45,50], PD subtype (i.e., akinetic rigid or tremor dominant), or FOG behaviors, such as complete akinesia, small stepping, and trembling in place [51], as well as slow stepping. External video recordings of participants’ SIP trials showed that some participants exhibited one, some, or all of the four types of FOG behaviors. This observation combined with the improved performance of participant-specific models compared to the general model in some participants support the use of personalized approaches for the treatment of FOG and gait impairment in PD.

### 4.4. Limitations

This study focused on sensors worn on the shanks, the most common body location for sensors aimed to detect FOG [41]. Future studies could look into sensor placements on other body locations. Although our logistic regression models achieved performance results similar to other IMU-based FOG algorithms [8,9,11,12,13,14,15,16,17,18,19,20,21,22,23,24,25,26,27,28,29,30,31,32,33,34,41], the training and testing sets were built using data from one trial of SIP from a small cohort of participants and were further reduced after balancing freeze and non-freeze steps. To increase the sizes of the training and testing sets, future models could include more trials of SIP from people who experience FOG in addition to data from people who do not experience FOG. The models’ freeze classifications were also binary (non-freeze and freeze). Future models could further discriminate freezes based on the variable FOG behaviors (i.e., complete akinesia, small stepping, trembling in place, and slow stepping).

## 5. Conclusions

The SIP task is a useful tool for eliciting FOG in the clinic and laboratory setting to study and treat FOG and gait impairment in PD. This study has demonstrated that gait parameters measured from wearable sensors reliably detected FOG during SIP, extending the accessibility of the SIP task for FOG assessment to the home environment. A general, logistic regression model built from the kinematic data revealed that three gait parameters together were overall the most robust predictors of FOG during SIP: arrhythmicity, swing time CV, and swing angular range. Participant-specific models revealed varying sets of gait parameters that best predicted FOG for each participant, highlighting variable FOG behaviors, and demonstrated equal or better performance than the general model for 6 out of the 10 participants. These results support the use of personalized approaches for the treatment of FOG and gait impairment in PD both inside and outside the clinical environment.

## Figures and Tables

**Table 1:** Participant demographics (*n* = 10).

Participant	Sex	Age (years)	Disease Duration (years)	UPDRS III Score	FOG-Q3 Score
1	M	65	6	25 *	1
2	M	60	6	48	3
3	M	44	7	56	0
4	M	57	11	55	1
5	F	61	12	36	2
6	F	61	16	38 **	3 **
7	F	75	8	27	N/A ***
8	M	56	11	29	1
9	F	69	14	38	4
10	F	71	8	32	4
**Average**		62.5	10.3	38.4	2.1
**Standard Deviation**		8.8	3.5	11.2	1.5

* and ** report scores from previous visit (* 1 month prior and ** 3 months prior), since no UPDRS III and/or FOG-Q3 score was recorded at the time of visit. *** no FOG-Q3 score was recorded at the time of visit and no other visits occurred.

**Table 2:** General logistic regression model performance.

**Area Under the Curve (AUC)**	0.81
**Accuracy**	0.84
**Sensitivity**	0.86
**Specificity**	0.81

**Table 3:** Coefficients of general and participant-specific gait parameters that best predicted freezing of gait during the stepping in place task.

		Peak Shank Angular Velocity	Stride Time	Swing Angular Range	Swing Time	Swing Time Coefficient of Variation	Asymmetry	Arrhythmicity	Freeze Index
General Model		−0.006		−0.06		0.894		1.076	
Participant-Specific Models	1	−0.578		−0.479	−0.558			0.318	
	2				0.627	0.544	0.181	1.831	
	3 *								
	4		0.065			0.466			
	5 *								
	6		0.619					3.104	
	7		0.505			0.399	0.341	0.633	
	8 *								
	9			−0.542	−0.118				
	10			−1.165	−2.474			1.293	

* participant-specific models calculated unstable coefficients due to small training and testing sets, thus no coefficients were reported.

**Table 4:** Participant-specific model performance.

Participant	Accuracy		Sensitivity		Specificity	
	General	Participant-Specific	General	Participant-Specific	General	Participant-Specific
1	0.96	0.96	1.00	1.00	0.92	0.92
2	0.69	0.81	0.63	0.88	0.75	0.75
3	0.60	0.55	0.50	0.40	0.70	0.70
4	0.75	0.63	0.75	0.75	0.75	0.50
5	0.75	0.75	1.00	0.75	0.50	0.75
6	1.00	0.75	1.00	1.00	1.00	0.50
7	0.67	0.83	0.83	1.00	0.50	0.67
8	1.00	0.50	1.00	1.00	1.00	0.00
9	0.93	0.95	0.90	0.95	0.95	0.95
10	1.00	1.00	1.00	1.00	1.00	1.00
Average	0.84	0.77	0.86	0.87	0.81	0.67
	Equal or better performance than the general model.					
	Lower performance than the general model.					

## Data Availability

The data presented in this study are available on request from the corresponding author.
